# Effects of Aquatic Therapy on Balance and Gait in Chronic Stroke: A Systematic Review with Exploratory Meta-Analysis

**DOI:** 10.3390/neurolint18040071

**Published:** 2026-04-17

**Authors:** Daniela Ivaldi, Gabriele Triolo, Roberta Lombardo, Carla Susinna, Giovanni Restuccia, Angelo Quartarone, Viviana Lo Buono

**Affiliations:** 1 Dipartimento di Patologia Umana dell’Adulto e dell’Età Evolutiva “Gaetano Barresi”, Universita degli Studi di Messina, Via Consolare Valeria 1, 98125 Messina, Italy; daniela.ivaldi@studenti.unime.it; 2 IRCCS Centro Neurolesi Bonino-Pulejo, SS113 Via Palermo C. da Casazza, 98124 Messina, Italy; gabriele.triolo@irccsme.it (G.T.); roberta.lombardo@irccsme.it (R.L.); carla.susinna@irccsme.it (C.S.); angelo.quartarone@irccsme.it (A.Q.); viviana.lobuono@irccsme.it (V.L.B.)

**Keywords:** aquatic therapy, balance, gait, rehabilitation, stroke

## Abstract

**Background**: Aquatic therapy is increasingly used in post-stroke rehabilitation, but its effects on balance and gait in the chronic phase remain variably reported. This systematic review aimed to evaluate the effects of aquatic therapy, alone or combined with land-based rehabilitation, on balance and gait in individuals with chronic stroke. **Methods**: A systematic search of PubMed, Embase, Scopus, and Web of Science was conducted between February and March 2026. Randomized controlled trials enrolling adults with chronic stroke and evaluating aquatic-containing interventions with quantitative balance and/or gait outcomes were included. Owing to clinical and methodological heterogeneity, the primary synthesis was narrative. An exploratory random-effects meta-analysis was additionally performed for post-intervention Berg Balance Scale (BBS) scores. **Results**: Thirteen randomized controlled trials involving 468 participants were included. Overall, aquatic therapy was associated with more consistent improvements in balance than in gait, while combined aquatic and land-based programs generally showed broader functional gains than land-based rehabilitation alone. In the exploratory meta-analysis, the primary pooled analysis of four studies favored aquatic-containing interventions for post-intervention BBS scores (MD = 3.69, 95% CI 2.69 to 4.69; *p* < 0.001), with no observed heterogeneity (I^2^ = 0%). **Conclusions**: Aquatic therapy may be a useful adjunctive rehabilitation strategy for improving balance in chronic stroke, whereas effects on gait appear more variable. These findings should be interpreted cautiously because the quantitative synthesis was exploratory and the overall evidence base remains heterogeneous and limited by small sample sizes and short follow-up.

## 1. Introduction

Stroke is an acquired brain injury, characterized by one or more lesions that impair normal brain function [1]. Clinically, it presents as a sudden onset of focal or global neurological impairment lasting more than 24 h or resulting in death, with no cause other than vascular origin [2]. Worldwide, ischemic and haemorrhagic strokes represent the second leading cause of death and the third leading cause of disability [3]. Post-stroke disability arises from neurological sequelae that affect multiple functional domains, including motor, cognitive, linguistic, behavioural, and sensory systems. The impact on activities of daily living (ADLs) depends on factors such as stroke subtype, lesion site, and lesion extent [1]. Motor impairments are the most frequent and disabling consequences of stroke. They typically manifest as contralateral hemiparesis or hemiplegia, initial hypotonia (often transient), and spasticity, which affects up to 90% of post-stroke subjects, causing resistance to passive movement and limiting voluntary activity. These motor deficits are often accompanied by abnormal reflexes [4,5]. Motor dysfunctions, particularly impaired tone and voluntary control, frequently lead to balance and gait impairments [6]. Balance is the ability to maintain the body’s center of mass within the limits of stability (LOS), in both static and dynamic conditions. Static balance refers to maintaining posture without movement, while dynamic balance refers to maintaining stability during motion or external perturbations [6]. Early detection and targeted intervention are essential to prevent worsening of balance dysfunction [7]. Hemiplegic gait is typically characterized by decreased walking speed and asymmetry in step length, swing time, and joint angles [8]. These alterations increase fall risk, restrict social participation, and reduce quality of life, underscoring the importance of targeted rehabilitation strategies [9,10]. Conventional physiotherapy, based on therapeutic exercise, remains the cornerstone of post-stroke rehabilitation. Interventions to restore motor control, tone, coordination, and postural stability have been shown to improve both balance and gait in the acute and chronic phases. Balance and weight-shift training, along with gait exercises, are among the most effective protocols [11]. However, for stroke individuals presented with severe motor limitations or those unable to tolerate land-based loading, complementary rehabilitation strategies may offer additional benefits [12]. Aquatic therapy has emerged as a promising rehabilitative treatment, leveraging the physical properties of water to create a supportive environment for movement [13]. Recent studies suggest that aquatic therapy can significantly improve balance and walking speed in chronic stroke, although current evidence is limited in quality and consistency [13]. Several systematic reviews have examined exercise-based interventions for balance and gait recovery after stroke, yet their findings are difficult to apply directly to the chronic phase or to the aquatic setting specifically. Reviews on land-based exercise therapy have demonstrated benefits for balance capacity in chronic stroke [11], and network meta-analyses have identified balance and gait training as effective rehabilitation strategies across the post-stroke continuum [6]; however, neither the aquatic environment nor its specific physical properties were addressed in those works. Reviews that have included aquatic therapy have generally pooled patients across different phases of stroke recovery—acute, subacute, and chronic—without stratifying findings by stroke chronicity, making it difficult to draw conclusions specifically applicable to the chronic phase, where recovery mechanisms and rehabilitation goals differ substantially [14,15]. Moreover, existing reviews have often pooled heterogeneous interventions without distinguishing between aquatic-only and combined aquatic–land protocols, and have devoted limited attention to the physical properties of water that may underpin the observed therapeutic effects. Therefore, an updated review focused on adults with chronic stroke and on balance and gait-related outcomes was warranted. The aim of this systematic review is to evaluate the effectiveness of aquatic therapy in improving balance and gait in individuals with chronic stroke, compared to conventional rehabilitation or other therapeutic strategies, and to determine its potential clinical value as a complementary component of long-term rehabilitation programs.

## 2. Materials and Methods

This systematic review was conducted in accordance with the Preferred Reporting Items for Systematic Reviews and Meta-Analyses (PRISMA) statement [16] and is registered with the International Prospective Register of Systematic Reviews (PROSPERO) under record number CRD420251110759. PRISMA checklist is available in the Supplementary Materials. 

### 2.1. PICO(S) Model

The research question was structured using the PICO(S) model (Population, Intervention, Comparison, Outcomes and Study) to systematically define the population, intervention, comparison, outcomes, and study design. The target population comprised adults (>18 years) with ischemic or hemorrhagic stroke in the chronic phase (defined as ≥6 months after stroke onset). The intervention involved structured aquatic therapy performed in a pool or hydrotherapy setting, delivered either as a stand-alone intervention or combined with land-based rehabilitation. For comparison, we considered land-based physiotherapy, usual care, no additional treatment, or alternative aquatic-based interventions. Primary outcomes were balance and gait, assessed through validated clinical scales and/or instrumented measures. Secondary outcomes, when available, included mobility-related measures, adverse events, and other rehabilitation-related outcomes. Regarding study design, only randomized controlled trials (RCTs) were considered.

### 2.2. Search Strategy

A systematic literature search was carried out from February to March 2026 across the PubMed, Embase, Scopus, and Web of Science databases. The search strategy included studies published from January 1990 to March 2026. The following search string was applied using appropriate Boolean operators: (“aquatic exercise program” OR “water exercise program” OR “aquatic therapy” OR “hydrotherapy” OR “water therapy” OR “water-based exercise” OR “aquatic Exercise”) AND (stroke OR hemiparesis OR hemiplegia). The search strategy was tailored to the specific syntax of each database.

### 2.3. Study Selection

All records retrieved from the databases were exported to Zotero and duplicates were removed through software-assisted and manual checking. Review articles, systematic reviews, and meta-analyses were excluded before title/abstract screening, as the review focused exclusively on primary interventional studies. Three reviewers (D.I., R.L., and G.T.) independently screened titles and abstracts according to the predefined eligibility criteria. Potentially relevant records were then assessed in full text. Disagreements were resolved by discussion, and when needed, a fourth reviewer (V.L.B.) made the final decision. The search identified 771 records: 120 were found in Web of Science, 242 in Scopus, 129 in PubMed and 280 in Embase. After removal of 441 duplicates and 74 review articles/systematic reviews/meta-analyses, 256 records underwent title/abstract screening. Of these, 199 were excluded, and 57 reports were sought for retrieval. Sixteen reports could not be retrieved, leaving 41 full-text articles assessed for eligibility. Twenty-eight full-text articles were excluded for the following reasons: non-randomized or otherwise inappropriate study design (n = 14), population not in the chronic stroke phase or mixed population not separable (n = 8), outcomes not relevant to balance/gait or different study aim (n = 1), and insufficient quantitative data for extraction (n = 5). Thirteen studies met the inclusion criteria and were included in the systematic review.

### 2.4. Inclusion and Exclusion Criteria

Studies were included if they:

(1) Were RCTs;
(2) Enrolled adults with chronic stroke (≥6 months after onset);
(3) Investigated a structured aquatic therapy intervention delivered in a pool or hydrotherapy setting, either alone or in combination with land-based rehabilitation;
(4) Included a comparator group (e.g., land-based therapy, usual care, or another aquatic intervention);
(5) Reported quantitative data for balance and/or gait outcomes.

Studies were excluded if they:

(1) Enrolled acute or subacute stroke patients, or mixed populations not separately analyzable;
(2) Were non-randomized, uncontrolled, or non-primary studies;
(3) Evaluated thermal/spa exposure, lake/sea therapy, or non-structured aquatic therapy;
(4) Did not report extractable quantitative data for balance and/or gait outcomes;
(5) Were conference abstracts, proceedings, editorials, letters, books, or case reports;
(6) Were not available in English full text.

These eligibility criteria ensured the inclusion of high-quality studies, thereby strengthening the methodological rigor of the systematic review and supporting evidence-based conclusions.

### 2.5. Data Extraction

Data were independently extracted by three reviewers (D.I., R.L. and G.T.) using a standardized data extraction form. Any discrepancies in data extraction were resolved by consensus or through consultation with a fourth reviewer (V.L.B.). The following information was collected from each study: first author, year, country, setting, sample size, age and sex distribution, time since stroke, intervention characteristics (including frequency, duration, session length, and water temperature), comparator, outcome measures, follow-up assessment, pre- and post-intervention values, between-group results, *p*-values, and adverse events or safety-related information when available. Information on attrition and handling of missing outcome data was extracted from each study and considered in the RoB 2 assessment. No imputation of missing means or standard deviations was performed. Studies lacking sufficient numerical information for quantitative synthesis were not pooled, and their findings were considered qualitatively when appropriate.

### 2.6. Risk of Bias Assessment

The methodological quality and risk of bias of included studies were assessed using the Cochrane Risk of Bias 2 (RoB 2) [17] tool for RCTs. Two reviewers (D.I. and G.T.) independently rated each study across the five RoB 2 domains: randomization process, deviations from intended interventions, missing outcome data, measurement of the outcome, and selection of the reported result. Discrepancies were resolved through discussion or consultation with a third reviewer (R.L.).

### 2.7. Statistical Analysis and Exploratory Meta-Analysis

Statistical synthesis was primarily narrative because of the clinical and methodological heterogeneity across studies, including differences in intervention content, comparator type, and outcome measures. However, an exploratory meta-analysis was additionally performed for post-intervention Berg Balance Scale (BBS) scores, as this was the most commonly shared balance outcome across the included trials. For the primary meta-analysis, only studies reporting standard BBS data and comparing aquatic-containing interventions with non-aquatic comparators were included. Mean differences (MDs) were calculated using post-treatment means, standard deviations, and sample sizes. In multi-arm trials, only one comparison was included in the primary analysis to avoid double-counting of the control group. Studies with marked baseline imbalance on the BBS were excluded from the primary pooling and considered in sensitivity analyses. A random-effects model was used for all pooled analyses. Heterogeneity was assessed using Cochran’s Q, tau-squared (τ^2^), and I-squared (I^2^). Ninety-five percent confidence intervals (95% CIs) and 95% prediction intervals (95% PIs) were reported. Meta-analyses were conducted using JASP software (Version 0.95.4.0; JASP Team).

## 3. Results

### 3.1. Included Studies

**Figure 1:**
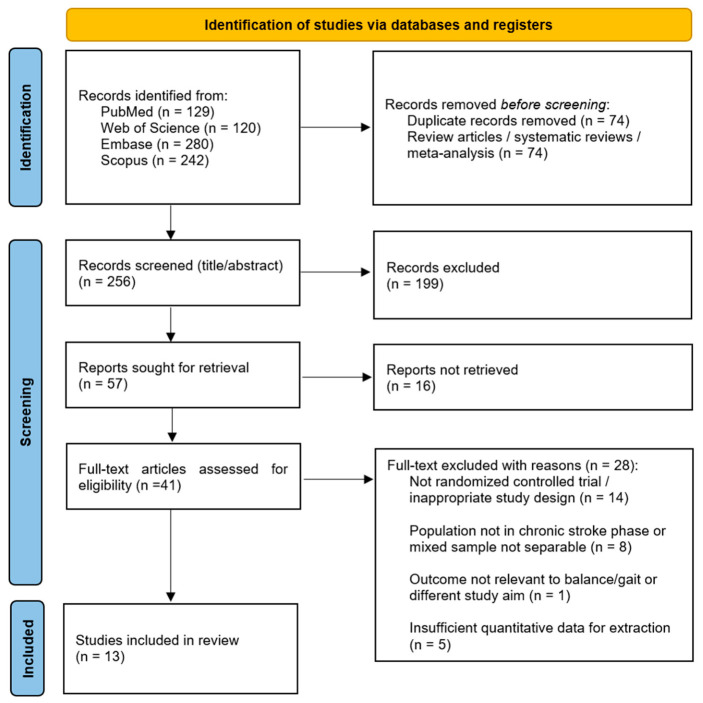
PRISMA flow diagram for research strategy.

This systematic review included 13 RCTs published between 2008 and 2021, for a total of 468 participants (Figure 1). Sample sizes ranged from 20 to 60 participants. Intervention duration varied from 4 to 12 weeks, with aquatic programs generally delivered 2 to 6 times per week. Across studies, pool water temperature was typically maintained between 27 °C and 36 °C. Detailed study characteristics and numerical outcomes are reported in Table 1 and Table 2.

### 3.2. Outcome Measures

The primary outcomes of interest were changes in balance and gait/mobility performance. Balance was assessed using validated clinical and functional measures, including the BBS, Functional Reach Test (FRT), Tinetti balance-related measures, tandem stance, single-leg stance, the Korean version of the Trunk Impairment Scale (K-TIS), the Postural Assessment Scale for Stroke Patients–3 Level version (PASS-3L), and the Berg Balance Scale–3 Level version (BBS-3L). Instrumented balance outcomes, when available, included LOS, center-of-pressure and stability indices, and Biodex-based balance measures. Gait and mobility outcomes included the Timed Up and Go (TUG), 10-Meter Walk Test (10MWT), 2-Minute Walk Test (2MWT), sit-to-stand-based measures such as the Five Times Sit-to-Stand Test (FTSTS), walking speed, and spatiotemporal gait parameters assessed through validated clinical tests or gait analysis systems. Secondary outcomes, when available, included activities of daily living, most assessed with the Modified Barthel Index (MBI), as well as spasticity, muscle strength, quality of life, and adverse events.

### 3.3. Results of the Narrative Synthesis

#### 3.3.1. Aquatic Therapy vs. Land-Based Therapy or No Additional Intervention

Five studies [13,21,22,25,28] compared aquatic therapy programs with conventional land-based therapy or no additional intervention. Overall, both groups generally improved over time, but aquatic therapy more often favored selected balance and mobility outcomes. Noh et al. [13] reported greater improvements with aquatic therapy in BBS and weight-shifting ability, while Zhu et al. [28] found significantly larger gains in the FRT and 2MWT, although between-group differences in BBS and TUG were not significant. Similarly, Saleh et al. [25] reported superior post-treatment results with aquatic motor dual-task training for instrumental balance indices and several gait parameters, and Aidar et al. [18] found significant group-by-time effects favoring the aquatic program on TUG, the 7.62 m walk, sit-to-stand performance, and balance. In Park and Roh [22], both groups improved under eyes-open conditions, whereas the aquatic group showed additional gains under eyes-closed conditions, suggesting greater benefit in more challenging sensory conditions.

#### 3.3.2. Combined Aquatic + Land-Based Therapy vs. Land-Based Therapy Alone

Three studies [19,20,23] assessed aquatic therapy combined with conventional land-based rehabilitation. Overall, the combined approach tended to produce broader improvements in balance, mobility, and function, although findings were not entirely uniform across studies. Kim et al. [20] reported greater between-group improvements in BBS, FRT, 10MWT, and TUG after underwater proprioceptive neuromuscular facilitation (PNF)-based training added to neurodevelopmental treatment. Park et al. [23] similarly found superior post-treatment scores in trunk control, balance, and activities of daily living in the land-based and aquatic trunk exercise (LATE) group, with between-group differences favoring the combined intervention for K-TIS, PASS-3L, BBS-3L, FRT, and MBI. By contrast, Eyvaz et al. [19] found no clear additional benefit of combining aquatic therapy and land-based exercise for most outcomes, and the improvement in BBS was significantly greater in the land-based group.

#### 3.3.3. Comparison of Aquatic, Combined, and Land-Based Therapy

Two three-arm trials by Pérez-de la Cruz [9,24] compared aquatic therapy alone, dry-land therapy alone, and a combined aquatic plus dry-land approach. In both studies, the combined intervention produced the most favorable overall pattern of results, while aquatic therapy alone generally performed better than dry-land therapy alone. In the 2020 trial [24], significant time-by-treatment effects were reported for Tinetti balance, Tinetti gait, Tinetti total score, and the 360° turn test, with improvements maintained at one-month follow-up in the aquatic and combined groups. In the 2021 trial [9], the combined group showed the clearest gains in BBS, TUG, FTSTS, and tandem stance, with benefits again persisting at one month.

#### 3.3.4. Comparison of Different Aquatic Interventions and Environments

Three studies compared different aquatic approaches rather than aquatic therapy against land-based rehabilitation [21,26,27]. Ku et al. [21] found that Ai Chi produced greater improvement than conventional aquatic therapyin anteroposterior dynamic balance and responder-based BBS change, although inter-group differences in gait parameters were not significant. Temperoni et al. [26] reported greater short-term improvement in BBS with the sequential preparatory approach than with conventional aquatic therapy, with benefits maintained at one-month follow-up. Vakilian et al. [27] found that both shallow- and deep-water exercise improved balance compared with control, particularly semi-dynamic balance, with no significant difference between the two aquatic depths.

#### 3.3.5. Balance Outcomes

Balance was the most frequently assessed domain across the included studies and was evaluated through both clinical scales and instrumented measures. The BBS was the most used clinical outcome, alongside the FRT, Tinetti balance-related measures, PASS, tandem stance, and single-leg stance, while instrumental assessments included limits of stability, center-of-pressure or stability indices, and Biodex-based balance measures. Overall, balance tended to improve after both aquatic and land-based interventions; however, aquatic-containing interventions more often showed broader or greater gains, particularly in studies targeting dynamic balance, weight shifting, or more challenging postural tasks [9,13,18,19,20,21,22,23,24,25,26,27,28]. At the same time, the magnitude and consistency of the between-group effects varied according to comparator type, intervention content, and measurement method. Detailed study-level quantitative results are presented in Table 2.

#### 3.3.6. Gait Outcomes

Gait and mobility outcomes were assessed using both clinical and instrumented measures, including TUG, 10MWT, 2MWT, FTSTS, gait analysis systems, and Biodex Gait Trainer parameters. In general, aquatic-containing interventions were associated with improvements in mobility and walking-related outcomes, although between-group differences appeared less consistent than for some balance measures. More favorable results were reported in studies using combined protocols, aquatic motor-task training, or more task-oriented aquatic interventions [9,20,23,24,25,28], whereas in other trials, improvements occurred in both groups without clear superiority on all gait outcomes [19,21]. Overall, the available evidence suggests a potential benefit of aquatic therapy for gait recovery in chronic stroke, although findings were more heterogeneous across studies and measures.

#### 3.3.7. Safety and Follow-Up

Safety reporting was inconsistent across studies. When explicitly reported, no significant treatment-related adverse events were described and compliance was generally high. However, several trials did not provide detailed safety data, and attrition was present in some studies. Only three studies included a one-month follow-up [9,24,26], all reporting at least partial maintenance of treatment-related gains. The absence of longer follow-up periods limits conclusions regarding durability of effects.

### 3.4. Results of Exploratory Meta-Analysis of Post-Intervention BBS Scores

**Figure 2:**
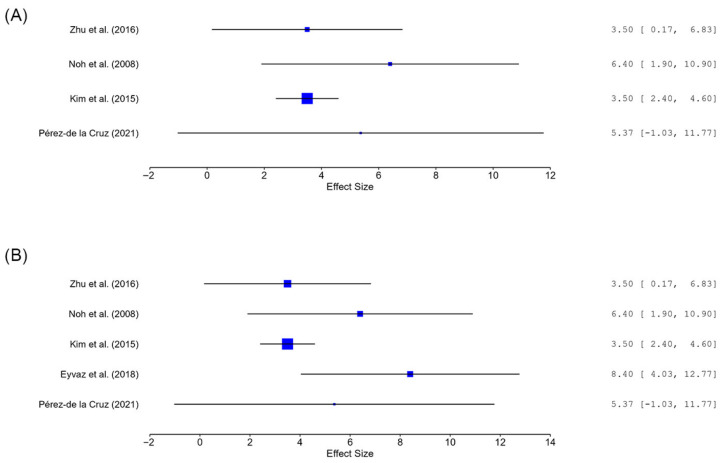
Forest plots of the exploratory random-effects meta-analysis of post-intervention Berg Balance Scale (BBS) scores comparing aquatic-containing interventions with non-aquatic comparators. (**A**) The primary analysis included four studies [9,13,20,28]; (**B**) sensitivity analysis additionally examined the impact of including a study [19] with marked baseline imbalance.

An exploratory random-effects meta-analysis was performed for post-intervention Berg Balance Scale (BBS) scores, as this was the most commonly shared balance outcome across trials. Figure 2 shows the forest plot of the meta-analysis.

#### 3.4.1. Primary Analysis

The primary analysis included four studies comparing aquatic-containing interventions with non-aquatic comparators [9,13,20,28]. The pooled mean difference favored aquatic-containing interventions (MD = 3.69, 95% CI 2.69 to 4.69; *p* < 0.001), with no observed heterogeneity (Q = 1.79, *p* = 0.618; I^2^ = 0%).

#### 3.4.2. Sensitivity Analysis

In sensitivity analysis, Eyvaz et al. [19], a study with marked baseline imbalance in BBS scores between groups was added to the pooled model. The direction of effect remained the same and continued to favor aquatic-containing interventions (MD = 4.72, 95% CI 2.81 to 6.62; *p* < 0.001), although heterogeneity increased (Q = 6.03, *p* = 0.197; I^2^ = 39.3%).

### 3.5. Risk of Bias

**Figure 3:**
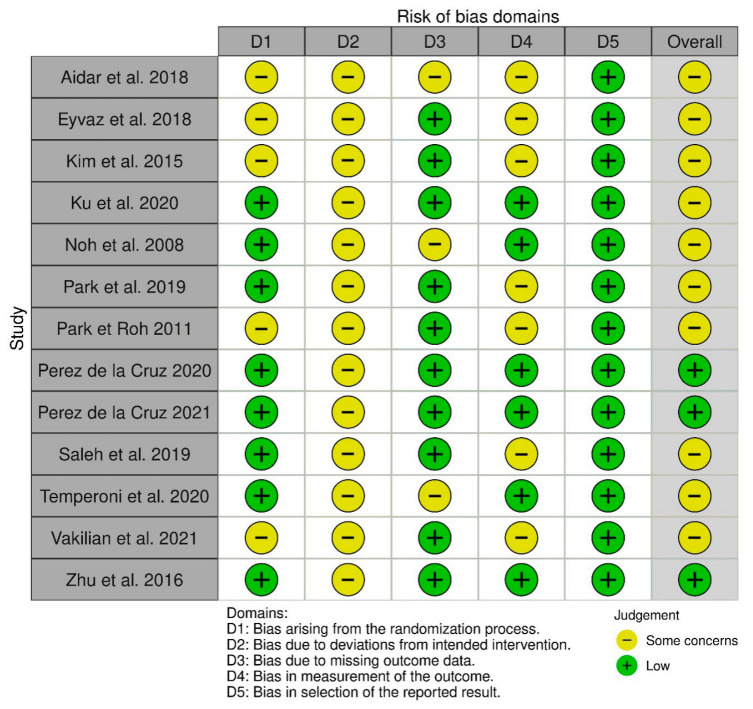
Visual representation of RoB 2.0 using risk of bias visualization tool (ROBVIS) [9,13,18,19,20,21,22,23,24,25,26,27,28].

The methodological quality of the included studies, as assessed using the RoB 2.0 tool [17], showed heterogeneity in the risk of bias, indicating variability in the internal validity among the trials, as illustrated in Figure 3.

Among the 13 studies included, 3 [9,24,28] were judged to have an overall low risk of bias, whereas the remaining 10 [13,18,19,20,21,22,23,25,26,27] presented methodological concerns in at least one domain, suggesting potential limitations in their internal validity. D1 (bias arising from the randomization process) was judged as low risk in most included studies, as they provided clear descriptions of the random sequence generation and allocation concealment procedures, with balanced baseline characteristics. However, in some studies, insufficient details on the randomization method and unexplained baseline imbalances led to ‘some concerns’. D2 (bias due to deviations from the intended interventions) was judged as presenting ‘some concerns’ across all included studies. This was mainly due to the lack of blinding of participants and personnel, which is inherently unfeasible in rehabilitation trials comparing aquatic versus land-based interventions. In most studies, therapists were not blinded, and no explicit measures were reported to control for potential performance or co-intervention biases. D3 (bias due to missing outcome data) was rated as low risk in most cases, owing to minimal or no loss to follow-up, well-documented reasons for attrition, and in some cases, the use of intention-to-treat analysis. Nonetheless, a few studies raised ‘some concerns’ due to the absence of ITT analyses and non-negligible missing data without appropriate handling strategies. D4 (bias in measurement of the outcome) was rated as ‘some concerns’ in the majority of included studies. This judgment was primarily based on the lack of clear information regarding whether outcome assessors were blinded to group allocation. Many of the outcomes were assessed using clinical scales that rely on the evaluator’s subjective judgment. In the absence of blinding, outcome measurement may have been unintentionally influenced by the assessor. Although some studies included more objective measures, the potential for detection bias could not be ruled out, thus justifying the overall concern in this domain. D5 (bias in selection of the reported result) was judged as low risk across all included studies. In most cases, outcomes relevant to this review were clearly reported, and no indications of selective outcome reporting were found. Even in studies without a preregistered protocol, the reported results were consistent with the stated aims and methods, and all prespecified outcomes appeared to be adequately presented. Overall, the methodological quality of included studies ranged from moderate to low, with common concerns related to blinding and heterogeneity of interventions.

## 4. Discussion

This systematic review suggests that aquatic therapy may be a useful rehabilitation approach for improving balance and, to a more variable extent, gait in individuals with chronic stroke.

Across the included RCTs, the most consistent benefits were observed for balance-related outcomes, including both clinical scales and instrumented measures of postural stability, whereas gait-related findings were generally more heterogeneous. In addition, studies investigating combined aquatic and land-based programs often reported broader functional improvements than conventional rehabilitation alone, supporting a possible complementary role of the two modalities [9,13,18,19,20,21,22,23,24,25,26,27,28].

The observed efficacy of aquatic therapy may be plausibly explained by the physical properties of water. Buoyancy reduces effective body weight and may facilitate the execution of movements that are more difficult to perform on land, particularly in individuals with impaired motor control. At the same time, hydrostatic pressure may enhance sensory input and postural stability, while viscosity provides natural resistance that can support muscle activation and motor control. Together, these characteristics create a therapeutic environment in which functional tasks can be practiced with reduced mechanical constraints and a lower perceived risk of falling [14,29,30]. In addition to mechanical properties, therapeutic water temperatures, typically maintained between 30 and 35 °C, may reduce spasticity and reflex hyperactivity, further supporting motor recovery [31]. Water temperatures promote muscle relaxation, reduce reflex activity, and may help decrease spasticity, improving movement quality and exercise tolerance [32,33]. Prior experimental work suggests that heat exerts an inhibitory effect on spinal reflex pathways, supporting its role in reducing muscle tone in neurological conditions [30]. In this context, hydrokinesis may be particularly beneficial for individuals with post-stroke spasticity, as the combined effects of buoyancy and warm water facilitate the execution of functional motor tasks.

Although gait outcomes improved in several trials, the effects appeared less consistent than those observed for balance. One possible explanation is that aquatic therapy may be particularly well suited to training postural control, weight shifting, and confidence during upright activities, whereas gait recovery depends more strongly on repetitive task-specific practice under full or near-full weight-bearing conditions [11]. Although buoyancy facilitates movement and reduces fear of falling, it may also partially reduce the loading and propulsion demands that characterize overground walking [29,30,34,35]. In this sense, the aquatic environment may better support the practice of balance-related components of mobility than the full mechanical demands required for terrestrial gait. Moreover, gait performed in water differs from gait on land because drag, turbulence, and altered loading conditions can modify spatiotemporal characteristics of movement. Altogether, these factors may help explain why balance-related gains were more consistent across studies, whereas transfer to land-based gait outcomes appeared more variable.

Studies comparing combined aquatic and land-based programs with conventional rehabilitation alone consistently reported greater improvements in balance and gait parameters, suggesting a potential synergistic effect between the two rehabilitation modalities [9,24]. From a clinical perspective, aquatic therapy may facilitate the early reacquisition of motor patterns by reducing mechanical constraints and fear of falling, while land-based training may reinforce the transfer of these gains to everyday functional activities. This complementary model supports the integration of aquatic therapy as an adjunct rather than a replacement for conventional physiotherapy. Beyond balance and gait, several studies also reported additional benefits, including improvements in muscle strength, reductions in spasticity, and enhanced psychological well-being [13,27].

A further clinically relevant aspect concerns trunk control. Since trunk stability is a key determinant of sitting balance, standing balance, and mobility after stroke, individuals with marked axial impairment may be particularly suitable candidates for aquatic rehabilitation [36]. In this population, trunk-focused exercise programs integrating both land-based and aquatic components have been shown to improve trunk control, balance, and activities of daily living, suggesting that the aquatic environment can be used not only for general conditioning but also for specific postural retraining [23]. This may be especially relevant for those patients who are unable to adequately stabilize the trunk against gravity on land, as buoyancy partially unloads the body while hydrostatic pressure provides circumferential support and enhanced sensory input, thereby facilitating earlier practice of upright alignment, weight shifting, reaching, and transitional tasks with less fear of falling [29,30,37].

An additional advantage of aquatic rehabilitation is that several therapeutic tasks commonly proposed on land can be translated into water while substantially modifying their mechanical demands. Exercises targeting trunk mobility, weight shifting, stepping, controlled rotational movements, and coordinated upper- and lower-limb activities can be practiced in water with progressive assistance or resistance, as reflected in stroke-related aquatic approaches such as Halliwick and Ai Chi-based interventions and in broader aquatic exercise literature showing that land-based exercise paradigms can often be reproduced in water with altered loading characteristics [13,15,38,39].

Aquatic environment also offers therapeutic opportunities that are difficult to reproduce on land with the same combination of unloading and safety. Because drag and turbulence generate velocity-dependent and multidirectional forces, water allows graded perturbation training, continuous resistance throughout the movement arc, and rotational challenges that may stimulate anticipatory and reactive postural adjustments in a distinctive way. This suggests that aquatic therapy should not be viewed merely as a lower-load version of land-based rehabilitation, but rather as a specific therapeutic setting in which novel movement experiences and task constraints can be created to challenge balance and motor control [29,34,35,40].

Safety findings should be interpreted with caution. Although no serious treatment-related adverse events were described in the studies that explicitly reported safety data, reporting was inconsistent across the evidence base and several trials provide little or no information on adverse events, tolerability, supervision procedures or safety monitoring. In addition, while attrition was reported in some studies, the reasons for withdrawal were not always clearly detailed, making interpretation more difficult. Importantly, one included study reported a fall-related hip fracture during follow-up [13]. Therefore, the current evidence should not be interpreted as demonstrating the absence of risk, but rather as reflecting inadequate and non-standardized safety reporting in this field. Future trials should systematically report adverse events, reasons for dropout, and monitoring procedures in order to better define the safety profile of aquatic therapy after stroke [9,21,28].

### Limitations

Despite these promising results, the current evidence should be interpreted with caution. The methodological quality of the included trials was variable, with several studies raising concerns related to allocation procedures, blinding, and sample size, which may have affected the internal validity of the reported findings. In particular, the inherent difficulty of blinding participants, therapists, and, in some cases, outcome assessors in rehabilitation trials may have increased the risk of performance and detection bias. Substantial variability across studies in intervention duration, training frequency, exercise protocols, and outcome measures, combined with generally small sample sizes ranging from 20 to 60 participants and an overall evidence base of only 468 participants across 13 studies, limits the statistical power of individual trials, hinders direct comparisons, and reduces the generalizability of the findings. Notably, although the therapeutic effects of warm water have been discussed in the literature and acknowledged in the included trials, the pool temperatures across studies ranged widely from 27 °C to 36 °C, with no consensus on the optimal thermal range for stroke rehabilitation. Similarly, session frequency varied from 2 to 6 times per week, and no study directly investigated the dose–response relationship between training frequency and functional outcomes. This variability makes it difficult not only to compare results across trials, but also to derive clinically actionable recommendations regarding the most effective intervention parameters. Moreover, only a small number of studies reported follow-up assessments beyond the immediate post-intervention period, limiting conclusions regarding the long-term sustainability of treatment effects, indeed only three of the thirteen included trials incorporated a post-intervention follow-up, and in all cases this was limited to one month, making it impossible to determine whether the observed gains in balance and gait are maintained over clinically meaningful time horizons. An additional limitation concerns the inconsistency of safety reporting across the included studies; several trials provided no information on adverse events, tolerability, or safety monitoring, which represents a notable gap. Without further systematic documentation of adverse events and compliance rates, conclusions about the safety profile of aquatic therapy should be interpreted with caution.

Beyond methodological heterogeneity, issues related to implementation and accessibility should also be considered when interpreting the clinical applicability of aquatic therapy. Recent qualitative evidence suggests that aquatic therapy is generally perceived as feasible by stroke survivors and caregivers, yet concerns regarding safety, supervision, and access remain important barriers to participation [41]. Aquatic therapy may therefore not be equally accessible to all patients, as its delivery is often dependent on the availability of appropriate pool facilities and on providers with specific qualifications or training in aquatic rehabilitation [42]. This indicates that the translation of positive trial findings into routine rehabilitation practice may depend not only on efficacy, but also on the availability of appropriate facilities, trained staff, and flexible program delivery [41,42]. Finally, although the risk of bias assessment identified only three studies with an overall low risk of bias, the implications of the remaining studies’ methodological weaknesses for the certainty of the overall evidence have not been fully accounted for in the conclusions. Findings from trials with high or unclear risk of bias may overestimate treatment effects, and the possibility of publication bias, given the small number of eligible trials and the general absence of null or negative results, cannot be excluded.

## 5. Conclusions

Overall, aquatic therapy appears to be a clinically relevant adjunctive option for chronic stroke rehabilitation, particularly when the therapeutic goal is to improve balance or when reduced weight-bearing and a safer practice environment are desirable. The most consistent benefits were observed for balance, whereas gait-related effects were more variable and may depend on intervention content and the integration with land-based task-specific training. However, recommendations for routine implementation should remain cautious because the available evidence is limited by small samples, heterogeneous protocols, inconsistent safety reporting, and short follow-up. To strengthen these findings, future large-scale, multicenter RCTs with standardized protocols and longer follow-up are needed to clarify the long-term effectiveness of aquatic therapy and better inform clinical decision making.

## Figures and Tables

**Table 1:** Characteristics of included studies.

Study	Sample Characteristics	Experimental Group	Control Group	Frequency and Duration	Main Outcomes	Follow-Up	Water Temperature
Aidar et al., 2018 [18]	36 (19 EG, 17 CG)	Aquatic exercise program	Began aquatic exercise program after 4 months	12 weeks, 2 sessions/week, 45–60 min/session	BDI, STAI I-II, BBS, TUG, Timed 7.62 m Walk, STST	/	27 °C
Eyvaz et al., 2018 [19]	60 (30 EG, 30 CG)	WBE + LBE	Only LBE	EG: 6 weeks: WBE 3/week + LBE 2/week; 60 min/session CG: 6 weeks: 5/week; 60 min/session	BBS, TUG, FIM, SF-36, Sportkat Balance Device, isokinetic measures	/	33 °C
Kim et al., 2015 [20]	20 (10 EG, 10 CG)	Underwater PNF coordination movement	Neurodevelopmental treatment	6 weeks, 5/week; 30 min/session	BBS, FRT, 10MWT, TUG	/	32–34 °C
Ku et al., 2020 [21]	20 (10 EG, 10 CG)	Ai Chi	Conventional water-based exercise	6 weeks, 3/week, 60 min/session	LOS, BBS, FMA, gait analysis	/	35 °C
Noh et al., 2008 [13]	25 (13 EG, 12 CG)	Ai Chi + Halliwick aquatic therapy	Conventional gym exercise	8 weeks, 3/week, 60 min/session	BBS, weight-bearing ability, muscle strength, MMAS gait	1-month post-intervention assessment	34 °C
Park and Roh, 2011 [22]	46 (23 EG, 23 CG)	Aquatic exercise	Only LBE	6 weeks, 6/week, 35 min/session	COP sway variables by Good Balance System	/	33–35 °C
Park et al., 2019 [23]	29 (14 EG, 15 CG)	Conventional physical therapy + LATE Program (LBE and WBE)	Conventional physical therapy	4 weeks, 5/week; 30 min LATE + 30 min conventional PT/day	K-TIS, PASS-3L, BBS-3L, FRT, MBI	/	33–34 °C
Pérez-de la Cruz, 2020 [24]	40 (13 EG1, 13 EG2, 14 CG)	EG1: Aquatic Ai Chi EG2: DLT + Ai Chi	DLT	12 weeks, 2/week, 50–60 min/session	Tinetti balance/gait, VAS, 360° Turn, single-leg stance, CS-30	1-month follow-up	34 °C
Pérez-de la Cruz, 2021 [9]	45 (15 EG1, 13 EG2 CG 17)	EG1: Aquatic Ai Chi EG2: DLT + Aquatic Ai Chi	DLT	12 weeks, 2/week	BBS, TUG, FTSTS, tandem stance	1-month follow-up	30 °C
Saleh et al., 2019 [25]	50 (25 EG, 25 CG)	Aquatic motor dual-task training	Land motor dual-task training	6 weeks, 3/week, 45 min/session	Biodex balance indices, Biodex Gait Trainer	/	33–35 °C
Temperoni et al., 2020 [26]	33 (18 EG, 15 CG)	Structured aquatic therapy protocol based on a progressive exercise program	Conventional aquatic training	4 weeks, 2/week, 45 min/session	BBS, MBI, Tinetti balance and gait, SS-QOL, MAS	1-month follow-up	30–32 °C
Vakilian et al., 2021 [27]	36 (12 EG1, 12 EG2, 12 CG)	EG1: Shallow-water exercise EG2: Deep-water exercise	No aquatic training	6 weeks, 3/week, 60 min/session	Biodex static and semi-dynamic balance measures	/	34–36 °C
Zhu et al., 2016 [28]	28 (14 EG, 14 CG)	Aquatic exercise + aquatic treadmill	Land-based exercise + treadmill	4 weeks, 5/week, 45 min/session	BBS, FRT, 2MWT, TUG	/	34–36 °C

Abbreviations: EG, experimental group; CG, control group; EG1, experimental group 1; EG2, experimental group 2; WBE, water-based exercise; LBE, land-based exercise; BDI, Beck Depression Inventory; STAI I–II, State–Trait Anxiety Inventory Forms I and II; BBS, Berg Balance Scale; TUG, Timed Up and Go; STST, Sit-to-Stand Test; FIM, Functional Independence Measure; SF-36, 36-Item Short Form Health Survey; PNF, proprioceptive neuromuscular facilitation; FRT, Functional Reach Test; 10MWT, 10-Meter Walk Test; LOS, Limits of Stability; FMA, Fugl–Meyer Assessment; MMAS, Modified Motor Assessment Scale; COP, center of pressure; K-TIS, Korean version of the Trunk Impairment Scale; PASS-3L, Postural Assessment Scale for Stroke Patients–3 Level version; BBS-3L, Berg Balance Scale–3 Level version; MBI, Modified Barthel Index; LATE, land-based and aquatic trunk exercise; DLT, dry-land therapy; VAS, Visual Analogue Scale; CS-30, 30-Second Chair Stand Test; FTSTS, Five Times Sit-to-Stand Test; SS-QOL, Stroke-Specific Quality of Life scale; MAS, Modified Ashworth Scale; 2MWT, 2-Minute Walk Test.

**Table 2:** Summary of the main results of the included studies.

Study	Main Balance Result	Main Gait/Mobility Result	Main Between-Group Finding	Safety
Aidar et al., 2018 [18]	Balance test improved from 42.3 ± 4.2 to 45.5 ± 4.1 in the aquatic group vs. 41.9 ± 6.0 to 41.4 ± 5.0 in controls	TUG improved from 13.1 ± 4.1 to 9.8 ± 3.9 vs. 13.8 ± 4.2 to 13.5 ± 4.1; timed 7.62 m walk improved from 9.4 ± 3.3 to 6.9 ± 4.2 vs. 9.1 ± 3.1 to 8.9 ± 4.1	Group × time favored aquatic exercise for TUG (*p* = 0.013, g = 0.91), 7.62 m walk (*p* = 0.031, g = 0.47), sit-to-stand (*p* = 0.042), and balance test (*p* = 0.012, g = 0.88)	3 aquatic dropouts; 4 controls not assessed post-treatment
Eyvaz et al., 2018 [19]	Both groups improved in BBS; however, BBS improvement was significantly greater in the land-based group	TUG and FIM improved significantly in both groups; no additional benefit of adding WBE to LBE for most outcomes	BBS change favored LBE alone (*p* < 0.05); only SF-36 vitality favored WBE + LBE (*p* < 0.05)	Safety not clearly reported
Kim et al., 2015 [20]	BBS improved 42.5 ± 1.1 to 45.1 ± 1.3 vs. 40.7 ± 1.5 to 41.6 ± 1.2; FRT improved 18.3 ± 1.1 to 20.4 ± 0.8 vs. 18.8 ± 0.9 to 19.4 ± 1.0	10MWT improved 14.6 ± 1.1 to 12.6 ± 1.7 vs. 14.9 ± 1.1 to 14.3 ± 0.9; TUG improved 18.4 ± 1.2 to 16.1 ± 1.6 vs. 18.5 ± 1.0 to 18.2 ± 1.0	Change scores favored underwater PNF for BBS (g = 2.68), FRT (g = 1.06), 10MWT (g = 1.20), and TUG (g = 1.51) (all between-group *p* < 0.05)	Safety not reported
Ku et al., 2020 [21]	Anteroposterior endpoint excursion increased 72.6 ± 13.0 to 105.0 ± 22.6 with Ai Chi vs. 80.2 ± 21.4 to 80.5 ± 21.2 in controls; both groups improved in BBS, but 6/10 vs. 2/10 exceeded MDC	Gait speed improved only after Ai Chi; no significant inter-group differences in gait parameters; FMA improved 22.0 ± 3.9 to 28.7 ± 4.2 vs. 21.7 ± 5.7 to 24.6 ± 7.7	Ai Chi favored for anteroposterior endpoint excursion (*p* = 0.001, g = 1.07), BBS responder analysis (*p* = 0.025), and FMA (*p* = 0.030)	No drop-out or adverse event reported
Noh et al., 2008 [13]	BBS improved 43.3 (5.2) to 50.9 (2.8) in the aquatic group; between-group improvement favored aquatic therapy	Gait (MMAS) improved in both groups, without significant difference between-group; knee flexor torque favored aquatic therapy	Between-group differences favored aquatic therapy for BBS (*p* = 0.032, author-reported Cohen’s d = 1.03), forward weight shift (*p* = 0.044, d = 1.14), backward weight shift (*p* = 0.031, d = 1.13), and knee flexor strength (*p* = 0.037, d = 1.13)	3 aquatic and 2 conventional dropouts; one fall-related hip fracture during follow-up
Park and Roh, 2011 [22]	Both groups improved static balance with eyes open; with eyes closed only the aquatic group improved consistently in X-speed, Y-speed and velocity moment	Mobility/gait outcomes were not primary endpoints	Between-group post hoc analysis showed significant differences in X-speed (*p* = 0.01, g = 0.65) and velocity moment (*p* = 0.01, g = 0.91); Y-speed also differed (*p* < 0.01)	Safety not reported
Park et al., 2019 [23]	PASS-3L improved 11.04 ± 2.09 to 13.04 ± 1.96 vs. 9.90 ± 2.99 to 10.80 ± 3.50; BBS-3L improved 16.71 ± 3.97 to 21.50 ± 3.94 vs. 19.87 ± 4.41 to 22.73 ± 3.60	FRT improved 23.42 ± 8.28 to 29.11 ± 7.80 vs. 28.58 ± 5.23 to 31.29 ± 5.62; MBI improved 64.07 ± 13.89 to 73.00 ± 13.00 vs. 56.73 ± 17.93 to 57.87 ± 17.92	Post-treatment between-group differences favored LATE for K-TIS (*p* = 0.001), PASS-3L (*p* = 0.034, g = 0.76), BBS-3L (*p* = 0.035), FRT (*p* = 0.045), and MBI (*p* = 0.001, g = 0.93)	1 early-discharge dropout
Pérez-de la Cruz, 2020 [24]	Tinetti total improved from 13.31 ± 4.2 to 18.62 ± 4.8 to 19.39 ± 4.9 in AQ + PT, from 13.07 ± 4.9 to 17.73 ± 5.1 to 17.73 ± 5.1 in AQ, and only modestly in PT; single-leg stance improved mainly in AQ + PT and AQ	360° turn and CS-30 improved markedly in AQ + PT and AQ versus PT	Significant time × treatment interaction for Tinetti balance (*p* = 0.011), Tinetti gait (*p* = 0.002), Tinetti total (*p* = 0.002; AQ + PT vs. PT: approx. g = 0.93; AQ vs. PT: g = 0.69), 360° turn (*p* < 0.001, g = 2.68 and 1.63, respectively), and single-leg stance (right *p* = 0.001)	No significant treatment-related adverse events; no dropouts
Pérez-de la Cruz, 2021 [9]	BBS improved from 27.31 to 33.08 in AQ + PT and from 27.73 to 43.80 in AQ, with minimal change in PT; tandem stance improved strongly in AQ + PT and AQ only	TUG improved from 19.06 to 13.77 in AQ + PT and from 19.13 to 12.80 in AQ; FTSTS improved from 24.91 to 11.92 in AQ + PT and from 25.40 to 15.67 in AQ	Significant time × treatment interactions favored both AQ + PT and AQ over PT for BBS (*p* < 0.001; AQ + PT vs. PT: g = 0.63; AQ vs. PT: g = 2.04), TUG (*p* = 0.004; g = 1.33 and 1.27, respectively), FTSTS (*p* < 0.001; g = 2.37 and 1.31, respectively), and tandem stance (*p* < 0.001; g = 1.32 and 1.79, respectively)	No adverse events reported
Saleh et al., 2019 [25]	Overall stability index improved from 3.38 ± 1.21 to 1.64 ± 0.45 vs. 3.40 ± 1.20 to 2.28 ± 0.92; APSI improved 2.66 ± 0.63 to 1.22 ± 0.14 vs. 2.72 ± 0.65 to 1.67 ± 0.78	Walking speed improved 47.92 ± 10.33 to 64.87 ± 11.54 vs. 48.04 ± 9.45 to 54.94 ± 10.24; affected-side step length and support time also favored water training	Aquatic dual-task training favored OASI (*p* = 0.02, g = 0.87), APSI (*p* = 0.03, g = 0.79), MLSI (*p* = 0.002), walking speed (*p* = 0.01, g = 0.90), affected step length (*p* = 0.03), non-affected step length (*p* = 0.01), and support time on affected side (*p* = 0.002)	Safety not clearly reported
Temperoni et al., 2020 [26]	BBS improved 40.8 ± 6.8 to 48.8 ± 6.4 to 51.4 ± 3.3 in SPA vs. 36.7 ± 11.1 to 40.7 ± 10.8 to 43.1 ± 13.0 in controls	Tinetti balance and gait also improved in both groups; SS-QOL favored SPA at follow-up	Between-group differences favored SPA for BBS at T1 (*p* = 0.01, g = 0.91), percentage improvement in BBS (*p* = 0.02), and SS-QOL at T2 (*p* = 0.03)	5 released before end of training; 4 additional dropouts at T2
Vakilian et al., 2021 [27]	Static balance post-training: 2.3 ± 1.0 (shallow), 2.1 ± 1.2 (deep), 3.5 ± 1.9 (control); semi-dynamic balance post-training: 0.6 ± 0.2, 0.5 ± 0.1, 0.9 ± 0.2	Gait outcomes not assessed	No significant difference between shallow and deep water; both intervention groups were superior to control for semi-dynamic balance (post-test *p* = 0.001; shallow vs. control: g = 1.45; deep vs. control: g = 2.44) and for static balance (post-test *p* = 0.037; shallow vs. control: g = 0.76; deep vs. control: g = 0.85)	Safety/adverse events not reported
Zhu et al., 2016 [28]	BBS improved from 38.5 to 49.4 vs. 37.9 to 45.9; FRT improved from 13.5 to 28.6 vs. 13.2 to 22.4	2MWT improved from 29.9 to 63.3 vs. 24.7 to 42.8; TUG improved from 29.7 to 21.2 vs. 30.4 to 23.6	Between-group improvements favored aquatic therapy for FRT and 2MWT (both *p* < 0.01, g = 1.60), whereas BBS and TUG differences were not significant despite moderate post-treatment standardized differences (BBS g = 0.76; TUG g = 0.86).	No significant treatment-related adverse events; no dropouts reported in the intervention program

Approximate post-treatment standardized effect sizes are reported as Hedges’ g, calculated from available group means, standard deviations, and sample sizes; positive values favor aquatic-containing interventions. For multi-arm trials, separate contrasts versus the dry-land/control group are shown. For Noh et al. [13], Cohen’s d was reported by the original authors. Effect size was not estimated for Eyvaz et al. [19] because of marked baseline imbalance. Abbreviations: AQ, aquatic therapy; AQ + PT, aquatic therapy plus physical therapy; PT, physical therapy; BBS, Berg Balance Scale; TUG, Timed Up and Go; FIM, Functional Independence Measure; SF-36, 36-Item Short Form Health Survey; FRT, Functional Reach Test; 10MWT, 10-Meter Walk Test;; FMA, Fugl–Meyer Assessment; MMAS, Modified Motor Assessment Scale; K-TIS, Korean version of the Trunk Impairment Scale; PASS-3L, Postural Assessment Scale for Stroke Patients–3 Level version; BBS-3L, Berg Balance Scale–3 Level version; MBI, Modified Barthel Index; CS-30, 30-Second Chair Stand Test; FTSTS, Five Times Sit-to-Stand Test; APSI, anteroposterior stability index; MLSI, mediolateral stability index; OASI, overall stability index; MDC, minimal detectable change; SPA, sequential preparatory approach; SS-QOL, Stroke-Specific Quality of Life scale; T1, post-intervention assessment; T2, follow-up assessment; 2MWT, 2-Minute Walk Test.

## Data Availability

No new data were created or analyzed in this study. Data sharing is not applicable to this article.

## Supplementary Materials

The following supporting information can be downloaded at: https://www.mdpi.com/article/10.3390/neurolint18040071/s1, PRISMA 2020 Checklist.

## Abbreviations

The following abbreviations are used in this manuscript:

ADLs	activities of daily living
BBS	Berg Balance Scale
BBS-3L	Berg Balance Scale–3 Level version
FRT	Functional Reach Test
FTSTS	Five Times Sit-to-Stand Test
K-TIS	Korean version of the Trunk Impairment Scale
LATE	land-based and aquatic trunk exercise
LOS	limits of stability
MBI	Modified Barthel Index
PNF	proprioceptive neuromuscular facilitation
RCTs	randomized controlled trials
TUG	Timed Up and Go
10MWT	10-Meter Walk Test
PASS-3L	Postural Assessment Scale for Stroke Patients–3 Level version
